# Efficacy and safety of Tianmabianchunzhigan in mild to moderate vascular dementia: Protocol of a randomized controlled IIa trial

**DOI:** 10.1097/MD.0000000000013760

**Published:** 2018-12-21

**Authors:** Jinzhou Tian, Jing Shi, Mingqing Wei, Ting Li, Jingnian Ni, Xuekai Zhang, Meng Zhang, Yang Li, Yongyan Wang

**Affiliations:** a Department of Neurology Centre, Dongzhimen Hospital, Beijing University of Chinese Medicine; b Peking Union Second Pharmaceutical Factory; c Institute of Basic Research in Clinical Medicine, China Academy of Chinese Medical Sciences, Beijing, China.

**Keywords:** Chinese medicine, randomized controlled trial, Tianmabianchunzhigan tablets, vascular dementia

## Abstract

**Background:**

Vascular dementia (VaD) is the 2nd most common subtype of dementia after Alzheimer disease. Currently, there are no medications approved for treating patients with VaD. Tianmabianchunzhigan (TMBCZG) tablet is an active ingredient extracted from *Gastrodia* that has been reported to improve memory and other cognition. And the TBMCZG has been approved clinical trial with patients with VaD by center for drug evaluation of China (CFDA). To evaluate the efficacy, safety, and tolerability of TMBCZG tablets in the treatment of mild to moderate VaD, we designed and reported the methodology for a 24-week, randomized, double-blind, parallel, placebo-controlled, multicenter trial.

**Methods:**

A total of 160 patients with mild to moderate VaD will be enrolled. After a 2-week run-in period, the eligible patients will be randomized to receive either TMBCZG high-dose group (TBMCZG 3 tablets, twice per day); TMBCZG middle-dose group (TMBCZG 2 tablets and placebo 1 tablet twice per day); TMBCZG low-dose group (TMBCZG 1 tablet and placebo 2 tablets, twice per day); placebo group (placebo 3 tablets, twice per day) for 24 weeks, with a follow-up 12 weeks after withdrawn drug treatment. The primary efficacy measurement will be the vascular dementia assessment scale-cognitive subscale and the Clinical Dementia Rating-Sum of the Boxes scale. The secondary efficacy measurements will include the mini mental state examination and activities of daily living. Adverse events will also be reported.

**Discussion:**

This randomized trial will be the 1st rigorous study on the efficacy and safety of TMBCZG tablets for treating cognitive symptoms in patients with VaD using a rational design.

**Trial registration:**

ClinicalTrials.gov NCT03230071. Registered on July 26, 2017.

## 1 Introduction

Vascular dementia (VaD) is a very frequent form of dementia, it had reported that dementia develops in around 15% to 30% of subjects 3 months after a stroke.^[1]^ At present, the treatment of VaD focuses on primary and secondary prevention strategies because randomized clinical trials in VaD have not been able to demonstrate clinically relevant symptomatic improvement. Although most have shown a small but significant benefit of cholinesterase inhibitors on cognition, the magnitude of this effect has been slight and benefits on global functioning, activities of daily living (ADL), and behavior have not been consistently reported; thus, cholinesterase inhibitors or memantine were not authorized for patients with VaD.^[2]^ Additionally, it has not yet been possible to establish disease-modifying effects in VaD syndrome.^[3]^ Thus, the development of an effective therapy for VaD is important.

Tianmabianchunzhigan tablet (TMBCZG) is an active ingredient extracted from *Gastrodia* (Tianma in Chinese) by Peking Union Second Pharmaceutical Factory. Preclinical studies have shown that TMBCZG can improve the learning and memory ability of rats with VaD and reduce the neuropathologic damage of model rats. A human tolerability test in a healthy person showed that the maximum tolerated dose of the drug was 84 mg/d, and the excessive adverse reactions were elevated urinary *N*-acetyl-β-d-glucosaminidase enzyme, hypotension, and diarrhea. In addition, toothache, sinus tachycardia, and elevated microalbuminuria for other suspected adverse events are further recommended for observation in expanded population samples. And the TBMCZG has been approved clinical trial with patients with VaD by center for drug evaluation of China (CFDA). To further investigate the safety, efficacy and effective dose of TMBCZG tablets in the treatment of mild to moderate VaD, we designed and report the methodology for a 24-week randomized, double-blind, parallel, multicenter study.

## 2 Methods

### 2.1 Study design, ethic, and setting

**Figure 1:**
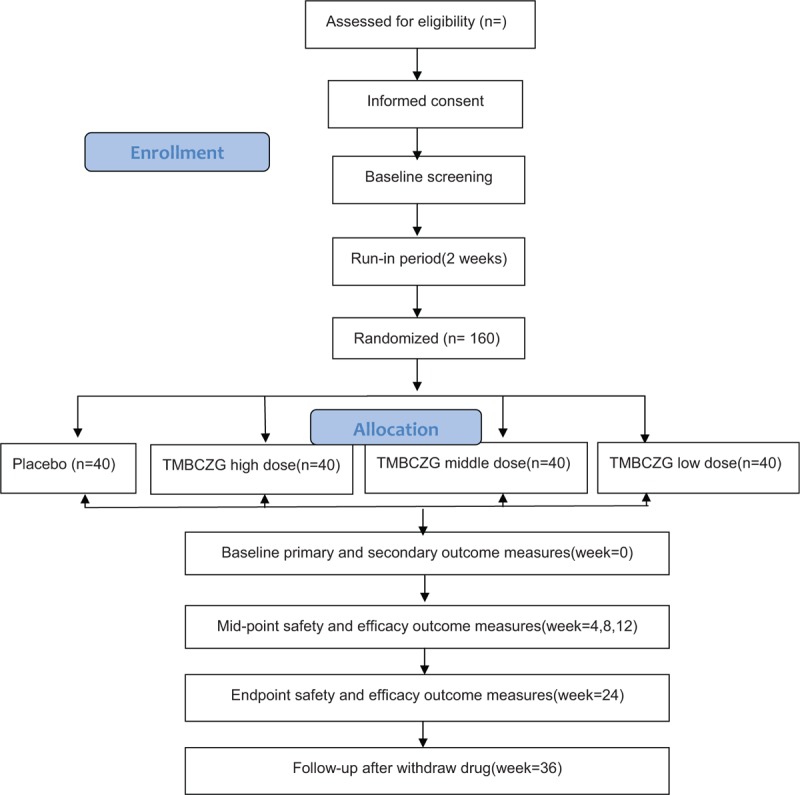
Study flow chart.

This ongoing study is designed as a randomized, double-blind, parallel, placebo-controlled, multi-central trial. It involves a single-blind run-in period using placebo only (2 weeks) and a double-blind treatment phase after randomization (24 weeks). It has been approved by the China Food and Drug Administration (code: 2012L02385). Ethics approval was requested and approved through the Institutional Review Board of Dongzhimen Hospital, Beijing University of Chinese Medicine (DZMEC-JG-2016-108) and has been registered with ClinicalTrials.gov (ClinicalTrials.gov NCT03230071) on July 26, 2017. The present study protocol (version 3, May 19, 2017) is designed in accordance with the Good Clinical Practice Guidelines and the principles of the Declaration of Helsinki. The protocol design is based on the guidelines of standard protocol items: recommendations for interventional trials (SPIRIT), and the study results will also be reported according to these guidelines. The patients and responsible caregivers will provide written informed consent. The study will be carried out at Dongzhimen Hospital and some other centers in China. A schematic of the trial protocol is detailed in Figure 1.

### 2.2 Participants

This trial will enroll both Chinese-speaking males and females, participants’ inclusion criteria are described as follows: Patients meeting the clinical diagnosis of probable VaD established according to the National Institute of Neurological Disorders and Stroke and the Association Internationale pour la Recherche et l’Enseignement en Neurosciences (NINDS-AIREN)were eligible to participate^[4]^: dementia defined by clinical core criteria; cerebrovascular disease, defined by history of stroke and through a magnetic resonance imaging (MRI) scan, including infarct in the main blood vessels, single strategic infarct (e.g., thalamus, angular gyrus, and basal forebrain), multiple lacunar infarcts, and/or extensive white matter damage surrounding ventricles (≥25% of all white matter area), hemorrhage^[5]^; a relationship between dementia and cerebrovascular disease, manifested, or inferred by the presence of one or more of the following: onset of dementia within 3 months following a recognized stroke; abrupt deterioration in cognitive functions; or fluctuating, stepwise progression of cognitive deficits.

Other inclusion criteria including:

1. mild to moderate dementia, defined with mini mental state examination (MMSE) score of ≤26 and ≥11^[6]^;
2. aged ≥55 and ≤80 years old; weighing of ≥45 and ≤90 kg;
3. adequate vision and hearing ability to complete all study tests;
4. have a stable caregiver;
5. can read simple articles and write simple sentences;
6. informed consent, signed informed consent by legal guardian.

They will also be assessed for the following exclusion criteria:

1. A medical history of other dementia types, like Alzheimer disease, frontotemporal dementia, Parkinson disease dementia, dementia with Lewy bodies, Huntington disease, etc;
2. Subdural hematoma, traffic hydrocephalus, brain tumor, thyroid disease, vitamin deficiency, or other diseases which can lead to cognitive impediment;
3. Major depression (Hamilton depression rating scale [HAMD] ≥17)^[7]^ or major anxious (Hamilton anxiety rating scale [HAMA] ≥12)^[8]^;
4. Subject cannot complete related test due to severe neurologic deficits, such as hemiplegia, aphasia, audio-visual disorder, and so forth;
5. Severe cardiovascular disease (severe arrhythmia, myocardial infarction within 3 months, New York Heart Association Functional Classification III and IV, systolic pressure ≥180 or ≤90 mm Hg);
6. Severe liver or kidney dysfunction (alanine aminotransferase or aspartate transaminase is more than 1.5 times the upper limit of normal, or serum creatinine is more than the upper limit of normal);
7. Uncontrolled diabetes (glycosylated hemoglobin is more than 2 times the upper limit of normal);
8. Asthma, chronic obstructive pulmonary disease, multiple neuritis, myasthenia gravis, and muscle atrophy;
9. Severe indigestion, gastrointestinal obstruction, gastric and duodenal ulcers, and other gastrointestinal disorders that can affect drug absorption;
10. A medical history of epileptic history, glaucoma, alcoholism, or psycho-substance abuse;
11. Subject has been taking cholinesterase inhibitors, memantine, nimodipine, or herbal medicine which have function to improve cognition in the past 1 month;
12. Use of sympathomimetic or antihistamines drugs within 48 hours before assessment;
13. Allergic constitution or allergic reactions to experimental drug;
14. According to the assessment of the investigator, subject cannot complete the study due to poor compliance or other reasons;
15. Subject is participating in other clinical trials or participated in the past 1 month.

### 2.3 Intervention

The TMBCZG tablet will be supplied by the Peking Union Second Pharmaceutical Factory. Each tablet weighs 0.1 g, which contains *Gastrodia* organic benzyl polyglycolate 14 mg. The TMBCZG tablets were produced in a single batch (batch number: 161101-kb, placebo batch number: 170201-kb) in strict compliance with standards of good manufacturing practice. During the 2-week placebo washout period, all patients will receive 3 placebo tablets twice times per day. During the double-blind, the patient will be randomized to TMBCZG high-dose group (TBMCZG 3 tablets, twice per day); TMBCZG middle dose group (TMBCZG 2 tablets and placebo 1 tablet twice per day; TMBCZG low-dose group (TMBCZG 1 tablet and placebo 2 tablets, twice per day); placebo group (placebo 3 tablets, twice per day). To preserve blinding, the placebo tablets have an identical taste and appearance to the TMBCZG tablets.

Concomitant use of anticonvulsants, antipsychotics, cholinomimetic drugs, anticholinergic agents, anti-Parkinson drugs, cholinesterase inhibitors, memantine, nootropic drugs, nimodipine, other cognition enhancers, traditional Chinese medicine tonic, or any drugs containing *Gastrodia* will be forbidden. The investigator must record the concomitant drugs, including the name of the drug, daily dose, reason for using, and date of termination.

### 2.4 Efficacy measurements

The primary efficacy measurements are changes of the vascular dementia assessment scale-cognitive subscale (VADAS-cog/17 items)^[9]^ and the clinical dementia rating-sum of the boxes (CDR-SB)^[10]^ scale from baseline to the 24th week after treatment. VADAS-cog is a revision of the ADAS-cog to be a better measure in vascular conditions,^[11]^ and a higher score indicates higher impairment. In addition to ADAS-cog, the VADAS-cog comprises additional frontal lobe tests reflecting attention, working memory, executive function, and verbal fluency.^[9]^ It was suggested that the VADAS-cog may be a more sensitive endpoint in patients with white matter load and vascular burden of the brain than ADAS-cog.^[12]^

The CDR-SB is a standard for disease grading in clinical studies of dementia and is used for overall endpoint assessment in clinical trials. It comprehensively assesses the cognitive and functional aspects of dementia patients, including memory, orientation, judgment and problem-solving skills, social affairs, family and hobbies, and personal cooking. The CDR-SB scores from 0 to 18 points, and a higher score indicates higher impairment.

The secondary efficacy measurements include the MMSE and ADL scale.^[6,13]^ The MMSE is used to assess the global cognition. ADL was used to measure the physical self-maintenance ability and instrumental ADL ability.^[13,14]^

### 2.5 Safety assessment

The safety assessment will include the following: vital signs: physical examination of vital signs, including rate of breathing, heart rate, and blood pressure; electrocardiography; the laboratory parameters included complete blood count, urine routine test, fecal routine and occult blood test, hepatic and renal function, coagulation function and electrocardiogram (ECG); and any adverse events that may occur, including the types of adverse events, time of occurrence, duration, treatment measures, and evaluation of the correlation between the tested drugs and the adverse event (positive, probable, possible, or not correlated); the severity of the adverse event (mild, moderate, and severe) must be evaluated.

### 2.6 Evaluation time point

The efficacy measurement will be assessed at baseline (week 0), at the midpoint (week 4 and week 12), and at the endpoint (week 24), 12 weeks after withdrawal (week 36).

The safety assessments will be evaluated at the baseline (week 0), at the midpoint (week 4, week 8, and week 12), and at the endpoint (week 24).

### 2.7 Sample size

There are no similar studies using TMBCZG tablets to treatment patients with VaD for the proposed study duration (24 weeks). The sample size was calculated based on the primary endpoints, VADAS-cog. It is hypothesized that the efficacy of TMBCZG in the treatment of mild to moderate VaD is similar to that of donepezil. Previous studies reported that the mean changes of ADAS-cog scores after 5 and 10 mg donepezil treatment were (5.75 ± 7.44) points and (2.25 ± 7.44) points, respectively. We assume that the placebo control group has a certain progression after 24 weeks’ treatment (△ = −0.25). The mean scores of the 3 groups of VADAS-cog scale improvement were set at equal intervals, that is, the changes before and after the scores of each group were −0.25, 0.75, 1.50, 2.25, and the variance of the 4 groups of VADAS-cog means 1.14. The standard deviation within is 7.44 points. For this phase IIa study, the 2-sided test level α = 0.20 and the power 1-β = 0.50. This clinical trial adopts a balanced design and uses nQuery Advisor 7.0 to calculate, 32 cases are needed for each group. Considering a greater rate of discontinuation of 20%, the sample size was increased to 39 per group to ensure adequate patients to complete the study. The total number of cases was 160 cases, that is, 40 cases in the placebo control group and 40 cases in the 3 dose groups of the TMBCZG tablets. Note also that because this study is a pilot efficacy study (phase IIa), a larger sample size to reach a higher degree of statistical power is not required.

### 2.8 Procedures

Two methods are being used to recruit participants with VaD. The 1st source of subjects is from memory clinics of different research centers, and the 2nd source is those who respond to advertisements published in local newspapers. Advertisements will be placed in local papers in surrounding the research centers; internet advertisements will be several website placed on different research centers.

Participants will undergo an initial face-to-face interview after written informed consent is obtained from both the participant and her informant, the participant's medical history and demographics will be taken with a semi-structured clinical interview, and participants will be estimated cognitive function using MMSE, HAMD, HAMA. Researchers will assess them against the study's inclusion and exclusion criteria, and the appropriate subjects will be initially selected. The subjects who decided to enroll will be assigned to run-in period. After 2 weeks run-in period, the subjects will be evaluated MMSE, VADAS-Cog, CDR-SB, ADL and vital signs, 12-lead ECG, laboratory tests, skulls MRI. Inclusion and exclusion criteria will be assessed again. And then subjects will be randomized to different treatment groups. Schedule of interventions and assessments at screening baseline, midpoint, and endpoint sessions is shown in Table 1.

**Table 1:**
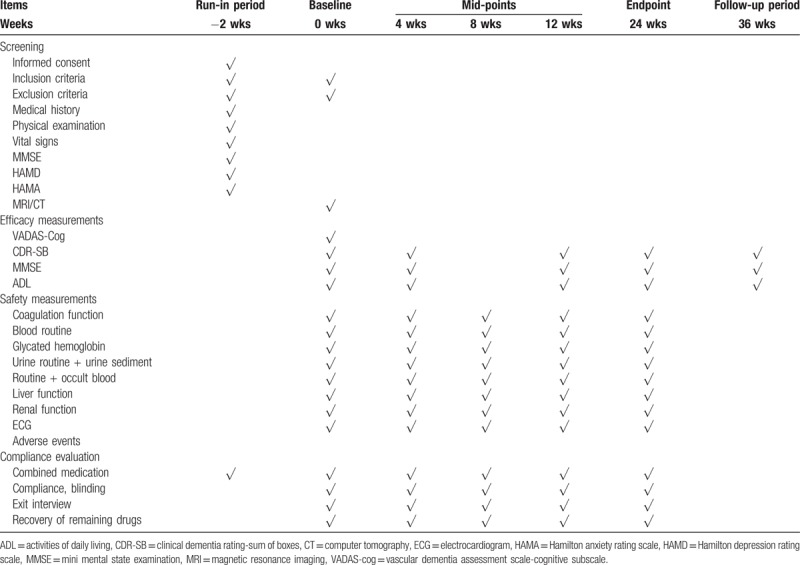
Schedule of interventions and assessments at screening baseline, midpoint, and endpoint sessions.

After baseline measurements, the first 4 weeks of medication will be dispensed. And the medication will be dispensed every 4 weeks. Participants will be required to return any unused medication at midpoint and endpoint; this will be used to determine compliance.

### 2.9 Study drug termination criteria

1) Prior to the maximum treatment period of 24 weeks, study drug will be discontinued if any of the following conditions are met:
2) Patient or informant request termination of study drug;
3) Patient or informant withdraws consent for the study;
4) Noncompliance with study drug as determined by the investigator (patients who have taken 80% or more of the expected number of tablets will be considered compliant);
5) Decision by the investigator that the study drug should be discontinued on safety grounds;
6) The reason for study drug termination should be recorded and any unused medication returned to pharmacy.

After the protocol is approved by the Ethics Committee, if modified, it must be reported to the Ethics Committee before execution and also recorded in the drug administration department.

### 2.10 Compliance strategy

To maximize subjects’ compliance, we will try to prevent dropouts by providing ongoing support to patients. A direct telephone line set up for this clinical trial will enable the study team to communicate personally with the patients. Participants will be offered reimbursement to cover travel expenses for their study visits: up to ¥100/visit will be provided and total travel expense will be up to ¥700. If a patient is lost to follow-up, we will call to ask the reason and attempt to schedule a meeting at the patient's convenience.

### 2.11 Randomization and blinding

In this trial, a complete randomization is adopted. Eligible subjects are randomly assigned to groups A, B, C, or D. The specific method is as follows:

1. The numbers of the observed cases are each labeled “No. 001-160.”
2. Using SAS 9.0, a biostatistics expert develops a computing procedure statement with set seeds, and a random number table is generated. Based on the table, a series of random numbers emerges and these are matched individually with each case number.
3. Subjects are divided into groups A, B, C, and D at the ratio of 1:1:1:1.
4. According to the case number and grouping, each subject is provided with the appropriate kit, with a drug number matching the case number.
5. Based on the case number, random number and grouping, emergency sealed envelopes are prepared and sent to the hospitals involved in the trial. The outside of each envelope is marked with the case number.
6. When a qualified subject is enrolled into the trial, the kit with the appropriate case number, in the order of subject enrollment, is provided.

The 1st blinding is case number matching groups, that is, group A, B, C, or D. The 2nd blinding is the disposal among the 4 groups. The blinding are sealed separately, duplicated, and stored in the research unit and the pharmaceutical factory. Once the blinding is broken, the patient will be managed as off-trial.

### 2.12 Statistical analysis

Statistical analyses will be conducted in 3 populations. The statistical analyses are planned to conduct in 3 populations. The intent-to-treat (ITT) population consist all randomized population who take at least 1 dose of medication and at least 1 primary efficacy evaluation on treatment. The fully evaluated (FE) population include all randomized patients who have receive at least 80% assigned 24 weeks’ double-blind medication with complete record of efficacy variable, with no major protocol violations. The safety set population included all randomized population and receive at least 1 dose of the study medication, with at least 1 safety record post baseline.

The efficacy analysis will be conducted with the ITT population and FE populations, for the VADAS-cog and CDR-SB will be analyzed by using the Last Observation Carried Forward method for the replacement of missing observations.

Multivariate analysis of covariance (ANCOVA) models that included baseline score as covariate, treatment, and center were used to assess differences between the treatment groups for linear efficacy measures. Categorical efficacy assessments were analyzed with a Cochran–Mantel–Haenszel test. The least squares mean changes from baseline scores to endpoint were presented for variables analyzed with the ANCOVA models. All values were 2-tailed, and all analyses were significant if the value was ≤0.05.

## 3 Discussion

*Gastrodia elata* (Tianma in Chinese), usually used to treat ischemic stroke, epilepsy, dizziness, and dementia for centuries in China in traditional Chinese medicine. TMBCZG is the active ingredient, which is extracted from *G elata*; studies have showed that gastrodin is effective in the treatment of bilateral common carotid artery occlusion-induced VaD via targeting Aβ-related protein formation and inhibiting autophagy and apoptosis of hippocampus neurons, ameliorates sub-acute phase cerebral ischemia reperfusion injury by inhibiting inflammation and apoptosis in rats^[15]^. However, there is no clinical trial on the treatment of VaD with TMBCZG tablets.

Studies showed that the most impaired cognitive domains in patients with VaD were attention/psychomotor speed, executive function, visuo-spatial (maze and structural tests in VADAS-cog), and language (animal fluency test). So we used the VADAS-cog scale as the primary efficacy measurement. And the neuropsychologic battery in this study covered these important domains comprehensively.

This ongoing randomized, double blind, parallel Group, placebo controlled, multicenter, 24-week clinical trial will be the 1st rigorous testing of TMBCZG tablets for the treatment of patients with VaD. Success in this clinical trial will provide evidence for the use of TMBCZG tablets in the treatment of VaD, and supply evidence of effective dose of TMBCZG in VaD.

## Author contributions

**Conceptualization:** Jinzhou Tian, Jing Shi, Mingqing Wei, Ting Li, Jingnian Ni, Xuekai Zhang, Yongyan Wang.

**Funding acquisition:** Jinzhou Tian, Mingqing Wei, Meng Zhang, Yang Li.

**Methodology:** Jinzhou Tian.

**Writing – original draft:** Jinzhou Tian, Jing Shi.

**Writing – review & editing:** Jinzhou Tian.
